# Irregularity—A Practical Case

**Published:** 1884-07

**Authors:** Charles P. Weinrich


ARTICLE III.

### IRREGULARITY.—A PRACTICAL CASE.

BY CHAS. P. WEINRICH, D. D. S.

Miss S. L. came to me for consultation. On examination I found some very irregularty and badly decayed teeth. The irregularity was entirely confined to the teeth of the superior maxilla. She seemed very much embarrassed by her appearance and the impediment in her power of speech, and inability to control the action of her lips. A badly decayed and pulpless central and lateral superior incisor added to the unsightly appearance. The masticating surface was small and imperfect. Being quite handsome and seemingly anxious about her appearance, etc., I felt certain that she would appreciate the results I aimed to accomplish. She was twenty years of age; the teeth large and very well formed. The case looked very unfavorable, indeed. The cuspids were exceedingly prominent. They rested upon the distal half of the labial surface of the lateral incisors. Their position was the main cause of the unnatural and noisome appearance of her mouth. When she laughed her lips would be drawn above the cuspid; the replacement of which required attention. While in repose the corners of her mouth were very prominent. She was very much elated when I assured her that this could be remedied. The prominence of the cuspid on the right allowed the first bicuspid to come within the thickness of thin blotting paper of touching the lateral incisor. The second bicuspid of this side was in its relatively proper position. The first molar was decayed below the margin of the alveolus; the second was badly decayed and the pulp diseased. On the left the cuspid was as prominent as the one on the right. The bicuspids did not antagonize with their fellows of the inferior owing to their inward inclination. They occluded inside and anteriorly to the bicuspids of the lower jaw. The first bicuspid was within one quarter the antero-posterior diameter of the cuspid of touching the lateral incisor, and the second in its relatively proper position. The first molar must have been extracted rather early, for the second had nearly attained the first molar's place in the arch. The palate between the bicuspids was quite high, and the rugue were prominent and had a crowded appearance. The teeth of the inferior maxilla were not regularly arranged; there being a slight crowding of the incisors and turning of bicuspids. This I perceived would do no particular harm, but on the contrary be gradually remedied by the pressure of the tongue and lip after relieving the pressure upon them by the superior incisors, owing to the crowding of cuspids, etc. The incisors and molars were the only teeth antagonized with their fellows of the inferior arch.

The patient came to me with the intention of having several teeth extracted; thinking that thereby to make room for the cuspids to attain their proper position in the arch; and having an artificial substitute made. But she finally consented to allow me to do what I thought best for the case.

After extracting the diseased roots of first molar on the right, I could, by judicious treatment, save the pulpless incisors and set in pieces of artificial teeth; for the central was minus its distal half of crown; and the lateral its mesial half. There was a slight discharge from these teeth of fetid matter. Her face had been swollen several months previous in consequence of an abscess.

She was very much gratified by the idea that I intended to do so much. She had been to several dentists quite recently who would have operated to her loss. One dentist wished to extract the cuspids, but she would not submit to the loss of a "front tooth." Another advised her to leave the unsightly cuspids as they were. He thought it not best to interfere with them.

Upon inquiry I learned that her parents, several years ago, had taken her to a dentist to have her teeth regulated. The dentist had extracted several of the deciduous set, and dismissed the patient with the idea that everything would go well now.

The patient stated that he gave them no instructions, but told them that the teeth would attain their proper position in the arch.

Still we grumble about the negligence of parents, and talk of educating the people.

When the irregularity is neither great nor complicated they will assume their proper position if the cause is removed between the tenth and ninteenth year. But the patient should be instructed to have them attended to during that period.

I found by measurement that by pushing the bicuspids of the left out and back they would antagonize properly, and there would be space enough to permit the cuspid to be drawn down into its proper place in the arch. By extracting the roots af the molar on the right and the bicuspids brought to their proper position, the cuspid would be permitted to assume its proper place.

I found that it was about as difficult to accomplish my desire, as it was to devise a means of regulating these teeth.

March 29th, 1883.—Took the impression. A few days after inserted a plate covering the hard palate and buccal teeth with the exception of those to be moved. Applied a jackscrew to each bicuspid on the left. She possessed the extent of masticating surface she previously had. It was necessary to separate the jaws somewhat to allow the bicuspids to be pressed out.

Although I met with several difficulties I did not expect, the teeth were moved, but not quite far enought back towards the molar.

During the use of this plate it was gouged out so as to permit the wedging of the bicuspids on the right; which, with the use of rubber wedges, were moved back somewhat. Wedges cut from block rubber are very effective. I did not know of its having been used for that purpose until a few months after I had employed it.

May 11th, 1883.—Removed this plate and substituted one covering the hard palate and extending around the tuberosity of maxilla. Four pieces of steel wire were bent to closely lay on the plate and pass between the bicuspids and bicuspid and cuspid, one each side. These springs were to push the bicuspids back, using hard plate, tuberiosities, and molars as fulcrum.

This apparatus was not a success. It moved the molars about as much as it did the pre-molars. If permitted to remain in the mouth it would have caused a great deal of mischief. The cuspids would not have sufficient space if the molars were brought forward. I found myself in quite a dilemma. I could not very safely make use of a fulcum. The teeth and palate did yield and would do so again. This yielding showed the practicability of Coffin's method of regulating. The molars had undoubtedly small roots and were not firmly attached. Therefore the impracticability of Farrar's method, as much as I know of it, in this case. There was no need of spreading the arch by a "Coffin plate," for there was sufficient room. I gave the applicability of Farrar's method to this case considerable though, and came to the conclusion that it could not be used. For the teeth that are generally firm and used as fulcums moved readily.

I next resorted to rubber bands and wedges. Stretched a band from molar to bicuspid thinking that perhaps the latter would move first and knowing that the molar would assume its original position if pressure or obstruction be removed. And a membrane having been irritated will absorb surroundings more readily than one not having been irritated. Having these facts in view, I took advantage of the cuspid and lateral, alternately, from which to wedge the bicuspids back, and succeeded.

June 21st, 1883.—Then made a plate covering the hard palate and tuberosities. This was the most effective apparatus I had used. Rubber bands were stretched from a hook on each side opposite the second bicuspids and stretched over the cuspids. This appliance began the same mischief the other one did, but fortunately, the cuspid were opposite their proper position,

July 16th, 1883.—Removed plate. Used ligatures and rubber bands and drew the cuspids into the arch. The lateral incisors, owing to the cuspids resting upon them, were pressed inwards, but they were brought out by the use of ligatures and bands, which were applied promiscuously to the buccal teeth as a fulcrum.

The ligatures and plate did not hinder the power of speech much.

The patient could not masticate with comfort. A privilege she had not enjoyed for some time.

August, 1883.—Thus the irregularity was removed. The bicuspids were retained in their new position by occlusion with the inferior corresponding teeth. The lateral and cuspids were retained by applying a clasp to a bicuspid and extending a piece of plate gold to labial surface of the cuspid and one to palatine surface of the incisors.

To replace the missing part of the lateral and central incisors on the right, after treatment, set in pieces, ground from artificial teeth, with "Weston's Insoluble Cement." To test the teeth and the fear of to great a contrast between the gold and the dead tooth structure, I thought it the best material.

Thus, I did all I could for the patient. The operation gratified me very much. Her parents and friends were very much pleased with her appearance and the success of the treatment. The change in her appearance was indeed very marked. Friends that had not seen her for some time spoke of the change in her countenance and could not think of the cause. Her mouth was perfectly healthy. She enjoyed a better masticating surface.

January, 1884.—I saw the patient. Her health was very good.—*Dental Register*,

